# Significant Impact of Previous Major Cardiovascular Events (MACEs) and Viremia on Risk of New MACEs in People Living with HIV on Antiretroviral Therapy

**DOI:** 10.3390/medicines13010004

**Published:** 2026-01-29

**Authors:** Caterina Candela, Alessia Siribelli, Tommaso Clemente, Riccardo Lolatto, Michele Bellomo, Vincenzo Stabile, Hamid Hasson, Vincenzo Spagnuolo, Antonella Castagna, Silvia Nozza, Camilla Muccini

**Affiliations:** 1 Infectious Diseases Unit, IRCCS San Raffaele Scientific Institute, Via Stamira D’Ancona 20, 20127 Milan, Italy; clemente.tommaso@hsr.it (T.C.); lolatto.riccardo@hsr.it (R.L.); bellomo.michele@hsr.it (M.B.); hasson.hamid@hsr.it (H.H.); spagnuolo.vincenzo@hsr.it (V.S.); castagna.antonella@hsr.it (A.C.); nozza.silvia@hsr.it (S.N.); muccini.camilla@hsr.it (C.M.); 2 Infectious Diseases Unit, Vita-Salute San Raffaele University, Via Olgettina 58, 20132 Milan, Italy; siribelli.alessia@hsr.it (A.S.); v.stabile@studenti.unisr.it (V.S.)

**Keywords:** PWH, ART, virological suppression, low-level viremia, MACEs

## Abstract

**Background:** Major cardiovascular events (MACEs) in people with HIV (PWH) may be partly related to antiretroviral therapy (ART) and persistent inflammation. The aim of the study was to evaluate the association between targeted variables and MACEs. **Methods:** Retrospective, single-center study conducted on PWH receiving ART between January 2010 and April 2024, classified according to HIV-RNA levels: virological suppression (<50 copies/mL), low-level viremia (50–200 or 200–1000 copies/mL), and non-suppression (≥1000 copies/mL). Viremia was considered as a time-dependent variable and by cumulative years in each category. A Cox proportional hazards model for multivariate time-to-event analysis assessed associations between virological status and MACEs. **Results:** We included 3349 PWH followed for a median time of 14 years (interquartile range, IQR 11.2–14.2). At baseline, 2794 (83.4%) were virologically suppressed, 189 (5.6%) and 90 (2.7%) presented 50–200 and 200–1000 copies/mL, respectively, and 276 (8.2%) were non-suppressed. During the follow-up, virological suppression was documented at least once in 3295 (98.4%), low-level viremia in 1579 (47.1%) with 50–200 copies/mL and 794 (23.7%) with 200–1000 copies/mL, and HIV-RNA > 1000 copies/mL in 844 (25.2%). Overall, 300 MACEs occurred, including 53 (17.7%) repeated events, with total incident rate of 0.00976 events per person-year. The risk of MACEs was significantly associated with previous MACEs (Hazard Ratio, HR 3.385, *p*-value < 0.001) and viremia > 1000 copies/mL at baseline (HR 2.209, *p*-value 0.039). Their onset was also significantly associated with greater age at baseline and years on ART, hypertension, diabetes, lower HDL, and higher triglycerides. **Conclusions:** PWH on ART with HIV-RNA > 1000 copies/mL at baseline and a previous MACE presented higher risk of developing MACEs.

## 1. Introduction

The remarkable success of antiretroviral therapy (ART) reshaped the human immunodeficiency virus (HIV) infection from a life-threatening condition to a manageable chronic disease, radically enhancing life quality and expectancy. In people with HIV (PWH), as HIV-related mortality declined, the clinical focus progressively turned to other long-term complications, with cardiovascular disease (CVD) emerging as a leading concern.

As already demonstrated, the risk of this population to develop major adverse cardiovascular events (MACEs) such as myocardial infarction, ischemic and hemorrhagic stroke, angina, and heart failure is double compared with people without HIV infection [1,2]. This etiology is not completely understood, but a growing body of evidence highlights a multifactorial nature, comprehending residual inflammation and chronic immune activation as central driver mechanisms [3,4], even among individuals who achieved virological suppression on ART and controlling for traditional risk factors [5,6]. On the other side, as ART is essential for achieving viral suppression, it can be associated with alterations in several metabolic processes, such as lipid metabolism, boosting the cardiovascular burden [7].

The current cardiovascular risk-prediction calculators used in the general population, such as the Framingham Risk Score (FRS) and the American College of Cardiology (ACC)/American Heart Association (AHA) Pooled Cohort Equation (PCE), tend to underestimate risk in PWH, where risk evaluation is notably challenging, resulting in insufficiently tailored cardioprotective recommendations [8,9,10]. In 2021, the European Society of Cardiology (ESC) introduced new models to estimate 10 year cardiovascular risk in Europe, named SCORE2 for PWH aged 40–69 years and SCORE2-OP for those older than 70 years [11]. In the absence of HIV-specific tools, the 2023 European AIDS Clinical Society (EACS) guidelines advise using these models as the primary approach for cardiovascular risk assessment in primary prevention among apparently healthy PWH without atherosclerotic cardiovascular disease, diabetes mellitus, chronic kidney disease, or familial hypercholesterolemia [12]. Consequently, strong primary preventive measures together with the creation of specific prediction tools are needed to ensure that the improvement in life expectancy due to ART is not hindered by morbidity and mortality from MACEs.

The aim of this study was to examine the multifaceted relationship between targeted variables like viremia, ART duration, demographic and clinical characteristics, and the incidence of MACEs in our cohort of PWH (Centro San Luigi, CSL cohort) over an extended period of follow-up.

## 2. Materials and Methods

This was an observational, retrospective, single-center study, conducted at Infectious and Tropical Diseases Unit of San Raffaele Scientific Institute in Milan, Italy, from January 2010 to April 2024.

### 2.1. Study Population and Design

The inclusion criteria included the following: age ≥ 18 years, confirmed HIV-1 infection, received ART for at least one year and at least one clinic visit between 1 January 2010 and 30 April 2024, and availability of follow-up data (≥1 year). Individuals with incomplete records or who did not provide informed consent were excluded.

Follow-up time accrued from the first eligible clinic visit within the study window to the earliest of MACE, death, loss to follow-up, or administrative censoring (30 April 2024).

Data were obtained from the Centro San Luigi (CSL) Cohort electronic medical records, which routinely record demographic, clinical, and laboratory information at visits scheduled approximately every 6 months. Standardized laboratory assessments included HIV-RNA quantification, CD4 and CD8 counts, lipid profile, glucose, and blood pressure.

HIV-RNA was modeled as a time-dependent exposure using clinically meaningful categories.

Individuals were classified into three categories, based on viremia levels: virological suppression was defined as HIV-RNA < 50 copies/mL, low-level viremia comprehended two subgroups with HIV-RNA 50–200 copies/mL and 200–1000 copies/mL, and virological non-suppression followed with HIV-RNA ≥ 1000 copies/mL.

The list of evaluated MACEs included myocardial infarction, ischemic stroke, angina pectoris, hemorrhagic stroke, peripheral ischemia, coronary reperfusion, and coronary bypass procedures. MACEs were identified through hospital discharge records and International Classification of Diseases, Ninth Revision (ICD-9) coding.

### 2.2. Endpoints

The study’s primary endpoint was to evaluate the association between the three different categories of viremia and the risk of MACEs. The secondary endpoint was to assess the association with the occurrence of MACEs and a range of demographic and clinical variables.

### 2.3. Variables

The following variables were considered in the analysis:

- Demographic variables: age and sex;
- HIV-related variables: duration of HIV infection, nadir and current CD4 cell count, CD8 cell count, CD4/CD8 ratio, and duration on ART;
- Treatment-related variables: exposure to different ART classes [Non-Nucleoside Reverse Transcriptase Inhibitors (NNRTIs), Protease Inhibitors (PIs), and Integrase Strand Transfer Inhibitors (INSTIs)], current regimen type, and cumulative time on each drug class;
- Cardiovascular risk factors and comorbidities: smoking status, body mass index, diabetes mellitus, hypertension, dyslipidaemia, history of chronic kidney disease, and family history of cardiovascular disease;
- Laboratory parameters: total cholesterol, low-density lipoprotein (LDL)-cholesterol, high-density lipoprotein (HDL)-cholesterol, triglycerides, fasting glucose, and blood pressure measurements.

### 2.4. Statistical Analysis

All demographic, clinical and virological data were recorded in electronic medical records and analyzed using R Statistical Software, version 4.2.2 (R Foundation for Statistical Computing).

Baseline participant characteristics were presented as median with interquartile range (IQR) or as frequencies with corresponding percentages (%). Viremia was assessed as a time-dependent variable and quantified in cumulative years since the start of follow-up. Hazard ratios (HRs) and *p*-values were calculated for risk factors, including age, duration on ART, lipid levels, and baseline viremia levels.

A Cox proportional hazards model for recurrent events (Andersen-Gill model) was used to perform a multivariate time-to-event analysis, assessing the association between virological categories and MACEs.

### 2.5. Ethical Approval

This study is based on clinical data collected in routine clinical practice. Ethical approval was obtained from the local Ethics Committee on 4 December 2017, protocol n. 34, and this approval is still in force. The protocol and informed consent form, which patients continue to sign, explicitly allows the use of clinical data for both retrospective and prospective analyses. Accordingly, the present work includes a retrospective evaluation of patient data from 1 January 2010 to 4 December 2017, as well as analyses performed thereafter, all of which fall within the scope of the approved protocol. Data were analyzed in anonymized form, and no additional procedures were performed for research purposes.

## 3. Results

### 3.1. Demographical and Clinical Characteristics of the Cohort

Among a total of 3349 participants, followed for a median time of 14 years (IQR 11.2–14.2), the median age was 46.1 years (41.8–50.8), with a predominance of 2532 males (75.6%).

The median duration of HIV diagnosis was 14.2 years (8.5–19.8), being on ART for a median of 11.6 years (5.9–14.2). The median CD4 count was 557 cells/µL (394–734) with the CD4 percentage of 26.3% (19.9–32.4) and the CD4/CD8 ratio of 0.55 (0.37–0.78). Lipid profiles indicated median HDL and LDL levels of 42 mg/dL (35–52) and 114 mg/dL (91–137), respectively, with median triglycerides 129 mg/dL (90–190). The median level of glucose was 87 mg/dL (81–96).

The median blood pressure systolic and diastolic values were 120 mmHg (114–130) and 80 mmHg (70–85), respectively. The median Body Mass Index (BMI) was 23.4 (21.3–25.8).

A summary of all demographic and clinical characteristics is provided in Table 1.

### 3.2. Interaction Between HIV RNA, CD4/CD8 Ratio, ART and MACEs

At baseline, 2794 (83.4%) people were virologically suppressed, 189 (5.6%) and 90 (2.7%) presented HIV RNA 50–200 copies/mL and 200–1000 copies/mL, respectively, and 276 (8.2%) were non-suppressed with HIV RNA ≥ 1000 copies/mL.

During the follow-up, virological suppression was documented at least once in 3295 (98.4%) cases, low-level viremia occurred in 1579 (47.1%) cases with HIV-RNA 50–200 copies/mL and 794 (23.7%) with HIV-RNA 200–1000 copies/mL, and HIV-RNA > 1000 copies/mL was detected in 844 (25.2%) cases.

The median CD4/CD8 ratio was inversely correlated with HIV RNA levels (Spearman ρ = −0.21, *p* < 0.001). In multivariate Cox regression, a lower CD4/CD8 ratio was significantly associated with an increased risk of MACEs (HR 1.35 per 0.1 decrease, 95% CI 1.05–1.74, *p* = 0.02), independently of age and baseline HIV RNA.

The cumulative duration of exposure to Non-Nucleoside Reverse Transcriptase Inhibitors (NNRTIs), Protease Inhibitors (PIs), and Integrase Strand Transfer Inhibitors (INSTIs), expressed per year, was 0.98 (0.928–1.027), 1.00 (0.946–1.047), and 0.99 (0.947–1.041), respectively.

Tenofovir disoproxil (TDF), emtricitabine (FTC), and ritonavir (RTV) were the most frequently prescribed at baseline in 1799 (53.7%), 1668 (49.8%), and 1393 (42.0%) cases, respectively. The most frequent ART combinations are listed in Table 2. In adjusted analyses, no regimen class was independently associated with MACEs once age, traditional risk factors, and viremia dynamics were included.

### 3.3. Distribution of MACEs

Overall, 300 MACEs were recorded, including 53 (17.7%) repeated events, with total incident rate of 0.00976 events per person-year. Particularly, 247 (7.4%) individuals experienced at least one MACE, while 36 (1.1%) had recurrent events.

Myocardial infarction was the most common event (161 cases, 53.7%), followed by ischemic stroke (64 cases, 21.3%), angina pectoris (9 cases, 3%), hemorrhagic stroke (5 cases, 1.7%), and peripherical ischemia (5 cases, 1.3%).

Coronary reperfusion and coronary by-pass were performed in 39 (24%) and 8 (2.7%) cases, respectively. Deaths occurred in 23 (6.3%) PWH.

All MACEs distribution is reassumed in Table 3.

### 3.4. Previous and New MACEs and Associated Variables

The risk of MACEs was significantly associated with previous MACEs (Hazard Ratio, HR 3.385, *p*-value < 0.001), and viremia ≥ 1000 copies/mL at baseline (HR 2.209, *p*-value 0.039).

**Figure 1:**
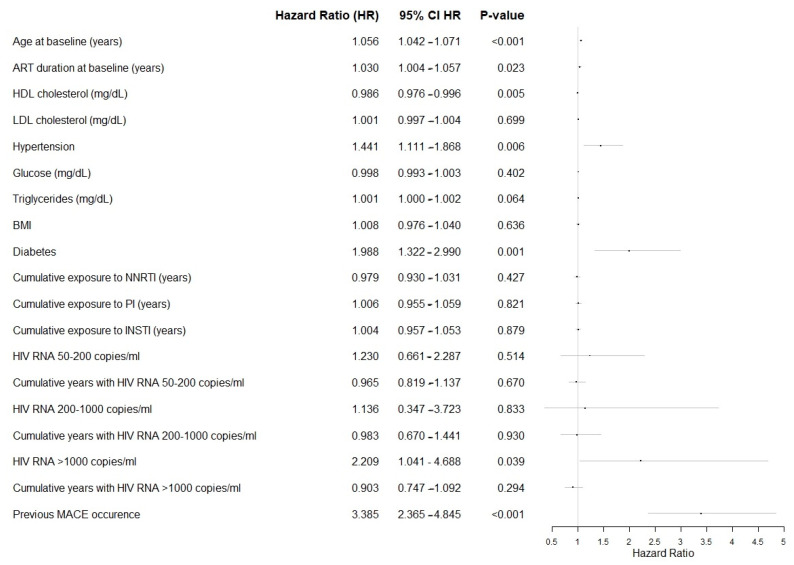
Forest plot of the targeted variables in the analysis, showing the effect size per unit increase. Abbreviations: ART, Antiretroviral Therapy; HDL, High Density Lipoprotein; LDL, Low Density Lipoprotein; BMI, Body Mass Index; NNRTI, Non-Nucleoside Reverse Transcriptase Inhibitors; PI, Protease Inhibitors; INSTI, Integrase Strand Transfer Inhibitors.

Among targeted variables, the onset of MACEs was also significantly associated with greater age at baseline and years on ART, hypertension, diabetes, lower HDL, and higher triglycerides, as described in Forrest Plot in Figure 1.

## 4. Discussion

This study provides significant insights into the multifactorial nature of MACEs among PWH receiving ART and the critical interaction with HIV RNA levels.

Our data highlight that, among PWH in routine care, sustained high-level viremia (≥1000 copies/mL) over time is the key modifiable predictor of MACEs, over and above age, traditional risk factors, and ART duration. This signal remained consistent across multiple specifications (time-dependent categories, continuous log10 modeling, spline sensitivity, and cumulative time in viremia states), emphasizing the clinical importance of durable suppression. While prior MACE strongly predicted recurrence, as expected in any cardiovascular population, the incremental information provided here is that viremia dynamics materially shape risk trajectories within PWH.

The viral suppression induced by ART certainly decreases the risk of HIV-related diseases and death, also reducing the risk of MACEs [13], but it may not fully control the chronic immune activation and the systemic inflammation, even in the context of virological suppression. Indeed, elevated inflammatory markers, including interleukin-6 and C-reactive protein, have been linked to leading to arterial inflammation, endothelial dysfunction and atherosclerosis, providing a mechanistic basis for increased risk of MACEs in PWH [14,15,16].

The stratification of viremia into the three distinct categories, virological suppression, low-level viremia, and virological non-suppression, permitted to analyze its role as a time-dependent variable and by cumulative years. Notably, high viremia ≥1000 copies/mL at baseline emerged as a significant predictor of MACEs, emphasizing the importance of achieving virological suppression. While high HIV RNA levels are clearly linked to mortality, AIDS-related events, and serious non-AIDS complications, the effect of low-level viremia on clinical outcomes remains uncertain [17]. Most evidence indicates an increased risk of subsequent virologic failure when viral load reaches 200 copies/mL or higher [18]. In our cohort, low-level viremia, especially with HIV RNA 50–200 copies/mL, frequently occurred during follow-up, although less pronounced in its impact compared to high-level viremia. Considering different findings by other studies where LLV has been demonstrated to be correlated to MACEs, our results cannot totally exclude the role of minimal viral replication to ongoing systemic inflammation and increased risk of CVD over time.

Importantly, the CD4/CD8 ratio emerged as an additional marker of cardiovascular risk, showing significant associations with both HIV RNA levels and the occurrence of MACEs. This highlights the value of incorporating immunological markers, beyond absolute CD4 counts, in cardiovascular risk stratification for PWH.

Nevertheless, the retrospective nature of the study prevents us from disentangling whether new MACEs are attributable solely to high viral load or to a broader definition of treatment failure (virological, immunological, or clinical). Our results indicate that viremia ≥1000 copies/mL remains the strongest independent predictor, even after adjustment for CD4 count and ART duration.

This study also highlighted the cumulative impact of ART duration on incidence of MACEs, as well as the role of frailty with greater age at baseline and other comorbidities such as hypertension, diabetes, lower HDL, and higher triglycerides. Indeed, the metabolic side effects of some ART regimes, especially protease inhibitors, warrant attention, because of their capacity of inducing dyslipidemia, insulin resistance, and fat redistribution, and consequently contributing to higher risk of CVD. An independent association between ART regimen class and MACEs after adjustment was not observed. Clinically, these findings argue for tight viral load monitoring, early management of persistent low-level viremia, and aggressive control of hypertension, diabetes, and dyslipidemia in PWH, particularly in those with a prior MACE.

The association between prior MACEs and increased risk of subsequent events underlines the importance of secondary prevention strategies. The observed risk of recurrent MACEs further underscores the importance of strict cardiovascular monitoring in PWH with a history of MACEs. The higher risk of CVD in PWH who have previously experienced such an episode can be explained not only by the individual familiarity and inadequate control of risk factors, but also by their enrollment in regular follow-up programs, which increases the likelihood of early diagnosis. Routine cardiovascular monitoring, rigorous risk factor management, and consideration of ART regimens with favorable metabolic profiles are critical components of care for PWH.

Classical cardiovascular risk prediction tools, like the previously mentioned Framingham Risk Score and the ACC/AHA Pooled Cohort Equations, may be limited in their ability to accurately assess risk among PWH. These models fail to account for HIV-specific factors, including the effects of chronic immune activation and ART, supporting the development of HIV-specific risk prediction tools that integrate both traditional and HIV-specific parameters to more accurately stratify cardiovascular risk. Furthermore, the lack of tailored cardioprotective recommendations for PWH underscores a critical gap in clinical practice.

The incorporation of designed primary prevention strategies, including lipid management, blood pressure control, and lifestyle modifications, is clearly needed, in addition to optimization ART strategies to minimize its metabolic side effects. A notable strength of this study is the extensive follow-up period, with a median of 14 years, which enabled a comprehensive evaluation of long-term cardiovascular outcomes. The large cohort size and the use of time-dependent variables further enhanced the robustness of the analysis.

However, the retrospective, single-center design may restrict the generalization of these findings. Moreover, the observational nature of the study prevents any causal inference between viremia and MACEs. Also, the underrepresentation of females could impair the capacity to assess the potential mediating effect of sex on the association between HIV and the cardiovascular risk. A further limitation is the lack of systematic data on HIV resistance profiles among patients with HIV RNA ≥ 1000 copies/mL. While resistance testing could provide additional insights into the mechanisms underlying persistent viremia, it was beyond the scope of the present study, which was focused on clinical and virological outcomes. The CSL Cohort already included a wide range of demographic, immunological, and virological variables, but future research incorporating resistance data may help to clarify this aspect.

## 5. Conclusions

Our study underlines the need of stringent cardiovascular monitoring and secondary prevention in PWH undergoing ART with HIV RNA > 1000 copies/mL at baseline and medical history of a previous MACE because of the high risk of developing new MACEs. These findings underscore the complex interplay between virological, pharmacological, and clinical factors in driving risk of MACEs among PWH and the importance of integrating cardiovascular health into the broader continuum of HIV care to optimize outcomes in this population.

## Figures and Tables

**Table 1:** Main characteristics at baseline.

Variables	N = 3349
Males (n, %)	2532 (75.6%)
Age (median, IQR)	46.1 (41.8–50.8)
Time from HIV diagnosis (median, IQR)	14.2 (8.5–19.8)
Duration on ART (median, IQR)	11.6 (5.9–14.2)
CD4 copies/mL (median, IQR)	557 (394–734)
CD4% (median, IQR)	26.3 (19.9–32.4)
CD4/CD8 ratio (median, IQR)	0.55 (0.37–0.78)
HDL mg/dL (median, IQR)	42 (35–52)
LDL mg/dL (median, IQR)	114 (91–137)
Triglycerides mg/dL (median, IQR)	129 (90–190)
Glucose mg/dL (median, IQR)	87 (81–96)
BP systolic mmHg (median, IQR)	120 (114–130)
BP diastolic mmHg (median, IQR)	80 (70–85)
Body Mass Index (median, IQR)	23.4 (21.3–25.8)

Abbreviations: ART, Antiretroviral Therapy; HDL, High Density Lipoprotein; LDL, Low Density Lipoprotein; BP, Blood Pressure; BMI, Body Mass Index.

**Table 2:** Main antiretroviral regimens at baseline.

ART Regimen	N
FTC + TDF + EFV	7068
TC + DTG	4532
FTC + TAF + RPV	4293
FTC + TDF + ATV + RTV	4273
FTC + TAF + BIC	3679
TC + ABC + DTG	3476
TC + ABC + ATV	3446
FTC + TDF + RPV	2544
DRV + RTV	2359
FTC + TDF + DRV + RTV	2287
FTC + TDF + ATV	2279
None	2028
FTC + TDF + LPV + RTV	1948
FTC + TAF + EVG + COBI	1661
FTC + TAF + DRV + COBI	1558
LPV + RTV	1471
FTC + TDF + NVP	1462
TC + ABC + NVP	1445
TC + ABC + ATV + RTV	1307
FTC + TAF + DTG	1256
TC + AZT + ABC	1200
RPV + DTG	1197
ATV + RTV	1181
FTC + TDF + RAL	1071

**Table 3:** Major cardiovascular diseases (MACEs) distribution.

Variables	N = 3349
Myocardial infarction (n, %)	161 (53.7%)
Ischemic stroke (n, %)	64 (21.3%)
Angina pectoris (n, %)	9 (3%)
Hemorrhagic stroke (n, %)	5 (1.7%)
Peripherical ischemia (n, %)	5 (1.3%)
Coronary reperfusion (n, %)	39 (24%)
Coronary by-pass (n, %)	8 (2.7%)
Deaths (n, %)	23 (6.3%)
Myocardial infarction (n, %)	161 (53.7%)
Ischemic stroke (n, %)	64 (21.3%)
Angina pectoris (n, %)	9 (3%)
Hemorrhagic stroke (n, %)	5 (1.7%)
Peripherical ischemia (n, %)	5 (1.3%)
Coronary reperfusion (n, %)	39 (24%)

## Data Availability

The data supporting the findings of this study were collected from routine clinical care of participants followed at the San Raffaele Scientific Institute and are stored in the CSL Cohort database. Due to ethical and privacy restrictions, the datasets are not publicly available. However, de-identified data may be made available from the corresponding author upon reasonable request and with appropriate institutional approval.
